# Enhancing Capsid Stability of a Foot-and-Mouth Disease Virus Vaccine Strain Through VP1-Directed Chimeric Design While Preserving Antigenicity

**DOI:** 10.3390/vaccines14050371

**Published:** 2026-04-22

**Authors:** Jong Sook Jin, Sun Young Park, Jae Young Kim, Giyoun Cho, Seung-A HwangBo, Jong-Hyeon Park, Young-Joon Ko

**Affiliations:** Center for FMD Vaccine Research, Animal and Plant Quarantine Agency, 177 Hyeoksin-8-ro, Gimcheon-si 39660, Republic of Korea; in75724@korea.kr (J.S.J.); sun3730@korea.kr (S.Y.P.); ivorikim@korea.kr (J.Y.K.); libretto@korea.kr (G.C.); hbsa230@kroea.kr (S.-A.H.); parkjhvet@korea.kr (J.-H.P.)

**Keywords:** foot-and-mouth disease virus, structural protein, VP1, stability, vaccine, immunization

## Abstract

**Background/Objectives**: The efficacy of inactivated foot-and-mouth disease virus (FMDV) vaccines depends on the structural integrity of the 146S virions. However, instability of 146S antigens during vaccine manufacturing and storage can compromise vaccine quality. Despite its high immunogenicity, the Korean serotype O strain O Jincheon (O JC) exhibits poor physical stability. **Methods**: To enhance antigenic stability while preserving strain-specific antigenicity, we engineered a VP1-substituted recombinant virus, (R) O1 M–O JC_VP1, by integrating the VP1 coding region of O JC into the O1 Manisa (O1 M) backbone. **Results**: The resulting chimeric virus exhibited significantly improved capsid stability, as demonstrated by an increased melting temperature and enhanced resistance to thermal stress, chloroform exposure, and long-term storage. Importantly, the recombinant antigen maintained its immunogenicity and induced antibody responses comparable to those induced by the parental O JC strain in vaccinated pigs. **Conclusions**: These findings demonstrate that VP1-direct chimeric engineering can improve capsid stability without compromising antigenicity and provide a practical approach for developing a stable FMDV vaccine.

## 1. Introduction

Foot-and-mouth disease (FMD) is a highly contagious viral disease affecting cloven-hoofed animals such as cattle, pigs, sheep, and goats. The causative agent, foot-and-mouth disease virus (FMDV), belongs to the genus *Aphthovirus* within the family *Picornaviridae* [1]. Owing to its rapid transmission, multiple serotypes, and capacity for long-distance spread, FMD poses a continuous threat to global livestock production and international trade. Outbreaks result in direct production losses and substantial economic consequences associated with trade embargoes, movement restrictions, and disease control measures [2]. In addition, large-scale outbreaks can lead to severe economic impacts due to mass culling, emergency vaccination, and disruptions in livestock supplies.

FMDV is a non-enveloped, positive-sense, single-stranded RNA virus with an icosahedral capsid composed of 60 copies of four structural proteins: VP1, VP2, VP3, and VP4. The outer capsid is formed by VP1–VP3, whereas VP4 is located internally [3,4,5]. These structural proteins collectively determine viral stability and receptor engagement, as they contain major neutralizing epitopes, including the G-H loop, which interacts with integrin receptors and is a principal target of protective antibodies [6,7,8]. Consequently, VP1 has been widely used for molecular epidemiology, antigenic characterization, and vaccine strain selection [9]. In addition to its antigenic role, the capsid structure as a whole plays a critical role in maintaining particle integrity, as inter-pentamer interactions and capsid cohesion directly influence the stability of intact virions.

Inactivated FMD vaccines are typically produced using purified 146S virions, which are intact immunogenic virus particles [10,11]. A persistent challenge in FMD vaccine production is maintaining the physical stability of 146S antigens. Antigen instability can lead to antigen degradation during production, formulation, storage, and transportation, thereby reducing immunogenicity and compromising vaccine efficacy [12]. In particular, dissociation of 146S particles into 12S subunits results in a significant loss of antigenic integrity, as the conformational epitopes required for neutralizing antibody induction are disrupted. Furthermore, such instability contributes to variability in antigen content, reduced shelf life, and increased dependence on cold-chain logistics, which pose challenges for vaccine distribution, especially in endemic regions.

Despite its strong immunogenicity, the Korean serotype O isolate O Jincheon (O JC) has been reported to show relatively poor physical stability, particularly during storage, despite exhibiting strong immunogenicity [13]. This discrepancy between antigenicity and stability highlights a significant challenge in vaccine strain selection, where highly immunogenic strains may not be suitable for large-scale vaccine production due to their inherent instability. Several studies have attempted to enhance FMDV stability by targeting amino acid substitutions in the capsid proteins. While mutation-based approaches can improve thermal stability, substitutions within or near key antigenic sites may alter antigenicity or affect vaccine matching. In addition, formulation-based approaches using stabilizing agents have been explored; however, these strategies cannot fully overcome the intrinsic instability of the viral capsid and often require extensive optimization for each vaccine formulation [14,15,16].

In this study, we evaluated a chimeric engineering strategy in which the VP1 coding region from the antigenically relevant O JC strain was introduced into the O1 Manisa (O1 M) backbone. As VP1 harbors major antigenic determinants, this chimeric design preserves O JC-specific antigenicity by incorporating the O1 M backbone, which exhibits relatively high structural stability among serotype O viruses [17]. We generated a recombinant virus, designated (R) O1 M–O JC_VP1, and characterized its particle morphology, physicochemical stability, and serological immunogenicity in pigs. Through this approach, we aimed to demonstrate that VP1-directed chimeric engineering can improve capsid stability without compromising antigenicity and provide a practical and scalable strategy for developing stable FMDV vaccine candidates, particularly for antigenically relevant but structurally labile strains.

## 2. Materials and Methods

### 2.1. Construction of Recombinant FMDVs

The recombinant virus (R) O1 M–O JC_VP1 was generated by replacing the VP1 coding region of the FMDV O1 Manisa backbone (O1 M; GenBank accession no. AY593823), and the corresponding VP1 sequence from the FMDV O Jincheon strain (O JC; GenBank accession No. 162590.1). An infectious cDNA clone was constructed in the pBluescript II SK(+) vector (Stratagene, La Jolla, CA, USA) by Gibson Assembly. Replacement of the VP1 region was performed using the GeneArt Gibson Assembly EX Cloning Kit (Thermo Fisher Scientific, Waltham, MA, USA) following the manufacturer’s instructions. The O1 M backbone and O JC VP1 insert were amplified with overlapping terminal sequences. These fragments were then incubated with the Gibson Assembly Master Mix containing the following enzymes: T5 exonuclease, DNA polymerase, and Taq DNA ligase. The resulting plasmid was transfected into BHK-21-T7-9 cells (RIKEN BioResource Research Center, Tsukuba, Japan), which stably expressed T7 RNA polymerase, using Lipofectamine 3000 (Invitrogen, Carlsbad, CA, USA). The recovered virus was subsequently passaged in ZZR (fetal goat tongue) cells (Fredrich-Loeffler-Institut (FLI), Greifswald-Insel Riems, Germany) and then adapted in BHK-21 cells (ATCC, Manassas, VA, USA).

### 2.2. Virus Cultivation, Inactivation, and Purification

Virus propagation and purification were performed to enable direct comparison of antigen stability between the recombinant and parental strains. BHK-21 cells were infected with (R) O1 M–O JC_VP1 and O JC wild-type viruses. Following infection, culture supernatants were harvested and clarified to remove cellular debris. Virus inactivation was carried out using binary ethylenimine (BEI; Sigma-Aldrich, St. Louis, MO, USA) and the resulting antigens were purified using polyethylene glycol (PEG 6000; Sigma-Aldrich) precipitation supplemented with 0.5 M NaCl (Sigma-Aldrich, St. Louis, MO, USA) followed by sucrose density gradient ultracentrifugation. Fractions corresponding to 146S particles were identified based on absorbance profiles and collected for subsequent stability and immunogenicity analyses.

### 2.3. Quantification of 146S Antigen by Size-Exclusion HPLC (SE-HPLC)

The content of intact 146S particles was determined using size-exclusion high-performance liquid chromatography (SE-HPLC) as previously described [18]. Separation was performed on a TSKgel G4000PWXL column (300 mm × 7.8 mm I.D.; Tosoh Bioscience, Tokyo, Japan) with a TSKgel PWXL Guardcol (40 mm × 6.0 mm). Elution was monitored at 254 nm using an Agilent 1260 Infinity II HPLC system (Agilent Technologies, Santa Clara, CA, USA), and 146S particles were quantified based on peak area measurements.

### 2.4. Transmission Electron Microscopy (TEM)

Purified wild-type O JC and (R) O1 M–O JC_VP1 viruses were adsorbed onto Formvar-coated copper grids, negatively stained with 1% (*w*/*v*) uranyl acetate, and visualized using a transmission electron microscope (H-7100FA; Hitachi, Tokyo, Japan).

### 2.5. Physical Stability Analysis of Purified 146S Antigen

The stability of purified 146S antigens was evaluated to assess the effect of VP1 substitution on capsid stability. The physical stability of the purified 146S antigens was evaluated using a particle stability thermal release assay (PaSTRy) and by examining their integrity after exposure to heat, chloroform, and refrigerated storage. PaSTRy was performed by mixing purified virus (1 μg) with SYBR Green II RNA dye (Thermo Fisher Scientific, Dartford, UK) in PBS and measuring fluorescence during a temperature ramp from 15 °C to 95 °C (0.5 °C every 10 s). Melt peak temperatures (T_m_, °C) were determined from the first derivative of fluorescence with respect to temperature (˗dRFU/dT). For the stress condition assay, purified antigens were subjected to thermal incubation (25 °C or 45 °C for 30 min), chloroform treatment (1:1, *v*/*v*; 5 min), or storage at 4 °C for up to 25 days. After each treatment, the remaining 146S antigen content was quantified by SE-HPLC and expressed as values relative to the untreated controls.

### 2.6. Vaccine Formulation

Vaccine formulations were prepared using purified 146S antigens derived from either the parental O JC or recombinant (R) O1 M–O JC_VP1 antigens (15 μg/dose) to enable direct comparison of immunogenicity. Each antigen was formulated with a combination of saponin (final 1%; Sigma-Aldrich) and aluminum hydroxide (final 10%; General Chemical, Parsippany, NJ, USA) as immunostimulatory components. The ISA 206 VG adjuvant (Seppic, Paris, France) was pre-warmed and mixed 1:1 (*v*/*v*) with the aqueous phase to a final volume of 2 mL/dose. Formulations were incubated at 20 °C for 1 h, protected from light, and stored at 4 °C until use.

### 2.7. Immunization of Pigs

Animal experiments were designed to compare immune responses elicited by the recombinant and parental antigens under identical conditions. Prior to vaccination, the pigs were screened for antibodies against FMDV, and only seronegative animals were included in this study. Twelve pigs were intramuscularly vaccinated with an FMD vaccine containing either the O JC wild-type antigen (*n* = 6) or the (R) O1 M–O JC_VP1 antigen (*n* = 6) with 15 μg of purified antigen. Booster immunization and sample collection were performed according to a defined schedule to monitor antibody responses over time.

### 2.8. Enzyme-Linked Immunosorbent Assay (ELISA)

FMDV-specific antibody responses were evaluated using a commercial solid-phase blocking ELISA kit (PrioCHECK™ FMDV Type O Antibody ELISA Kit; Thermo Fisher Scientific, Waltham, MA, USA), according to the manufacturer’s instructions. Briefly, serum samples were incubated in antigen-coated microtiter plates, and bound antibodies were detected using an horseradish peroxidase (HRP)-conjugated monoclonal antibody specific for FMDV type O. Following additional incubation and washing, substrate solution was added and the reaction was stopped. Optical density (OD) was measured at 450 nm using a microplate reader. Results were expressed as a percent inhibition (PI), and samples with a PI value ≥ 50% were considered positive.

### 2.9. Virus Neutralization Test (VNT)

The virus neutralization test for FMDV antibodies was performed according to the WOAH guidelines [19]. Serum samples were heat-inactivated at 56 °C for 30 min, serially diluted two-fold starting from 1:4, and added to wells of 50 μL each. Each dilution was mixed with 100 TCID_50_ of FMDV and incubated at 37 °C for 1 h. LFBK cells, derived from porcine kidney and provided by the Agricultural Research Service, USDA (Plum Island Animal Disease Center, Orient Point, NY, USA), were added at 0.5 × 10^6^ cells/mL. The plates were sealed and incubated at 37 °C with 5% CO_2_ for 2–3 days. The neutralizing titer was calculated as log_10_ values using the Spearman–Kärber method [20].

### 2.10. Statistical Analysis

Statistical analyses were performed using GraphPad Prism (version 9; GraphPad Software, San Diego, CA, USA). Statistical significance between the groups was determined using an unpaired *t*-test. Differences were considered statistically significant at *p* < 0.05, whereas non-significant results were indicated as “ns”.

## 3. Results

### 3.1. Generation and Morphological Characterization of Recombinant Virus

**Figure 1:**
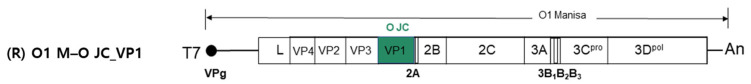
Schematic representation of the recombinant (R) O1 M–O JC_VP1 virus. The VP1 coding region of the O1 Manisa (O1 M) strain was replaced with the corresponding sequence from the O Jincheon (O JC) strain, while all other genomic regions were retained from the O1 M backbone. The substituted VP1 region is highlighted in green.

**Figure 2:**
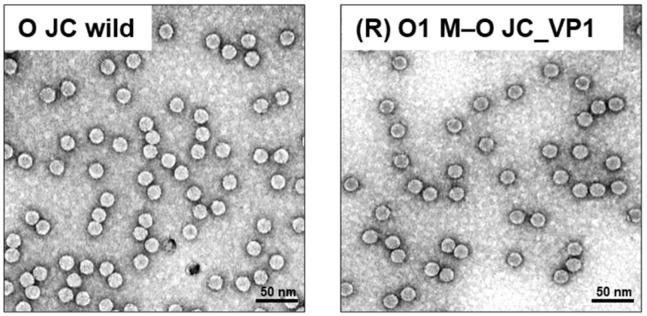
Morphological analysis of purified FMDV particles. Purified O JC wild-type and (R) O1 M–O JC_VP1 viruses were negatively stained with 1% uranyl acetate and visualized by transmission electron microscopy (TEM). The scale bar indicates 50 nm.

A recombinant FMDV, designated (R) O1 M–O JC_VP1, was generated by substituting the VP1 region of the O1 Manisa backbone with that of the O JC strain (Figure 1). The infectious recombinant virus was recovered following transfection and serial passaging of BHK-21 cells. Sequence analysis confirmed correct replacement of the VP1 coding region without mutations. TEM analysis revealed icosahedral particles with diameters of approximately 25–30 nm in both O JC wild-type and (R) O1 M–O JC_VP1 viruses (Figure 2).

### 3.2. Physicochemical Stability of Purified 146S Antigens

**Figure 3:**
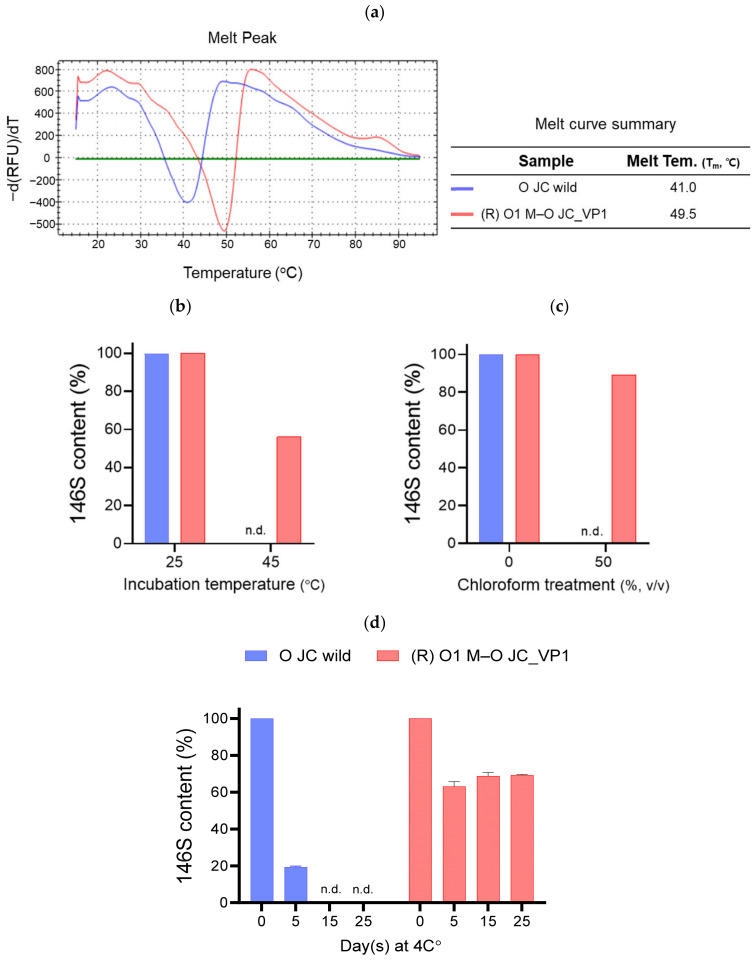
Comparative evaluation of the physical stability of O JC wild-type and (R) O1 M-O JC_VP1 viruses. (**a**) Thermal stability was evaluated by a particle stability thermal release assay (PaSTRy) using the RNA-binding dye SYBR Green II; the first derivative of fluorescence is shown, and melting temperatures (T_m_, °C) are summarized in the table. (**b**–**d**) The stability of purified 146S antigens was further determined by quantifying 146S content by high-performance liquid chromatography and presenting the results as relative values after (**b**) incubation at 25 °C or 45 °C for 30 min, (**c**) chloroform treatment (purified virus: chloroform = 1:1, *v*/*v*) for 5 min, and (**d**) storage in PBS at 4 °C for 0, 5, 15, and 25 days. n.d, not detected.

To determine whether VP1 substitution influenced capsid stability, the physicochemical stability of purified 146S antigens was compared using PaSTRy and SE-HPLC (Figure 3). In PaSTRy, (R) O1 M–O JC_VP1 exhibited a higher melting temperature (T_m_ = 49.5 °C) than O JC wild-type (T_m_ = 41.0 °C) (Figure 3a). Following incubation at 45 °C for 30 min, the 146S antigen of O JC wild-type was not detected, whereas (R) O1 M–O JC_VP1 retained 56% of 146S content relative to its 25 °C control (Figure 3b). After chloroform treatment (purified virus: chloroform = 1:1, *v*/*v*; 5 min), the 146S antigen of O JC wild-type was not detected, whereas (R) O1 M–O JC_VP1 retained 89% of its 146S content (Figure 3c). During storage in PBS at 4 °C, 146S antigen of O JC wild-type decreased over time and was not detected by day 15, whereas (R) O1 M–O JC_VP1 maintained 63–69% of the 146S content by day 25 (Figure 3d).

### 3.3. Humoral Immune Responses in Vaccinated Pigs

**Figure 4:**
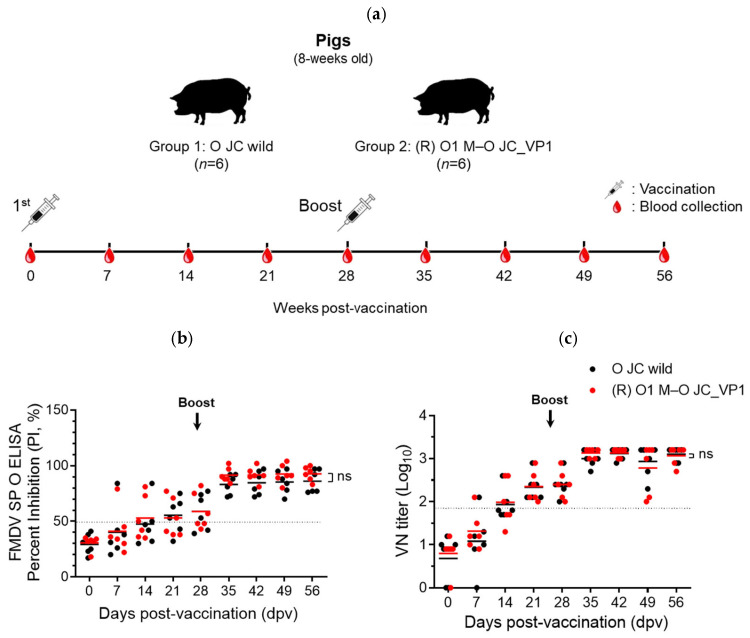
Immunogenicity evaluation of O JC wild-type and (R) O1 M–O JC_VP1 vaccines in pigs. (**a**) Study design and sampling schedule. Eight-week-old pigs were immunized (day 0) and boosted (day 28) with O JC wild-type (Group 1, *n* = 6) or (R) O1 M–O JC_VP1 (Group 2, *n* = 6) vaccines; blood was collected at the indicated time points. (**b**) Serological responses measured by FMDV structural protein (SP) O ELISA and expressed as percent inhibition (PI, %). A PI value of ≥50% (dotted line) was considered to indicate a positive result. (**c**) The homologous virus-neutralizing (VN) antibody titers were measured using the virus neutralization test. A titer ≥ 1:45 (1.65 log_10_) (dotted line) was considered to indicate a positive result. ns, not significant. Bars represent mean values.

To evaluate the immunogenicity of the vaccine formulations, humoral immune responses were determined in pigs vaccinated with wild-type O JC or (R) O1 M–O JC_VP1 antigens. Pigs were vaccinated on day 0 and received booster immunization 28 days post-vaccination (dpv) (Figure 4a). Structural protein O ELISA analysis demonstrated that mean antibody levels in both vaccinated groups exceeded the seropositivity cutoff value (percent inhibition (PI) ≥ 50%) from 21 dpv (Figure 4b). In the O JC wild-type group, mean PI values were 29.2 at 0 dpv, 40.2 at 7 dpv, 47.5 at 14 dpv, 55.3 at 21 dpv, 59.0 at 28 dpv, 83.0 at 35 dpv, 84.5 at 42 dpv, 85.3 at 49 dpv, and 86.0 at 56 dpv. Antibody responses increased progressively over time and were further enhanced following booster immunization. No statistically significant differences in mean PI values were observed between the two groups at any time point (*p* > 0.05). Consistent with the ELISA results, Virus neutralizing antibody titers against the O JC wild-type virus increased in both groups following vaccination (Figure 4c). At 21 dpv, mean VN titers reached 2.3 log_10_ in the O JC wild-type group and 2.4 log_10_ in the (R) O1 M–O JC_VP1 group. Following booster immunization, VN titers further increased, peaking at approximately 3.0–3.1 log_10_ at 35–42 dpv in both groups. Neutralizing antibody titers remained stable after booster immunization, with no statistically significant differences observed between the two vaccine groups (*p* > 0.05).

## 4. Discussion

Inactivated FMD vaccines rely on the preservation of intact 146S virions, which represent the immunogenic conformation of the capsid and are closely correlated with vaccine potency. However, many field-relevant strains exhibit pronounced particle lability, which leads to the dissociation of 146S particles into 12S subunits during processing, formulation, and storage. Such instability can reduce the effective antigen content, increase batch-to-batch variability, and impose strict cold-chain requirements. Consequently, improving the physical stability of vaccine antigens remains an important objective in FMD vaccine development. The South Korean isolate O JC is a relevant example, as it induces strong humoral immune responses, but exhibits poor physical stability under conditions relevant to vaccine manufacturing and refrigerated storage [21,22].

Our experimental design was guided by preliminary analyses identifying the viral capsid regions associated with the instability of the O JC strain. As shown in Supplementary Figure S1, chimeric viruses were generated by individually substituting VP1, VP2, or VP3 from the O JC into the genetically stable O PanAsia2 backbone [23], and their thermostability was evaluated by PaSTRy analysis. This experiment indicates that the instability associated with the O JC strain is primarily linked to VP2, as the substitution of O JC VP2 into the O PA2 backbone results in the largest reduction in melting temperature. In contrast, the substitution of O JC VP1 had minimal impact on capsid stability, suggesting that VP1 contributes relatively little to the intrinsic instability of the O JC capsid. Therefore, we selected the O1 Manisa (O1 M) backbone–a well-characterized vaccine strain with proven structural stability [17]—to host the O JC VP1 sequence, hypothesizing that the stable O1 M capsid environment would mitigate the destabilizing influence of O JC VP2/VP3.

These observations are consistent with previous studies demonstrating that VP2 plays a key role in maintaining capsid integrity [15,24]. Residues within VP2 contribute to inter-pentamer interactions along the two-fold symmetry axis, which are essential for stabilizing the capsid structure and preventing the dissociation of virion particles [25,26,27]. Therefore, disruption of these interactions can significantly affect particle stability and capsid cohesion.

Based on these findings, we hypothesized that introducing the antigenically important VP1 region of O JC into a physically stable capsid backbone could preserve strain-specific antigenicity while improving particle stability. To test this, we generated (R) O1 M–O JC_VP1 by replacing the VP1 coding region of the O1 M backbone with that of O JC. The resulting recombinant virus produced morphologically typical icosahedral particles comparable to those of the parental virus, indicating that VP1 substitution did not interfere with particle assembly or overall capsid structure.

VP1 substitution significantly improved physicochemical stability across multiple experimental conditions, including heat, chemical, and storage conditions. The recombinant antigen exhibited a higher melting temperature than the O JC wild-type antigen in PaSTRy analysis and retained detectable levels of intact 146S particles following thermal stress, chloroform exposure, and prolonged storage at 4 °C. Notably, under stress conditions, in which the 146S particles of the O JC wild-type antigen were completely degraded, the recombinant antigen maintained measurable levels of intact virions. These findings suggest that the chimeric VP1 design enhanced capsid robustness and improved resistance to environmental stresses relevant to vaccine production and storage.

Importantly, these improvements in stability did not compromise immunogenicity. Pigs vaccinated with the (R) O1 M–O JC_VP1 antigen exhibited serological responses comparable to those induced by the parental O JC antigen, as demonstrated by both SP ELISA and virus neutralization tests. In addition, a vaccine containing O JC-derived VP1 has been shown to protect against viral infections, as demonstrated by protection against challenges with multiple FMDV topotypes [21]. These results indicate that substitution of the VP1 region preserves the antigenic properties required to induce effective humoral immune responses.

Various strategies have been explored to improve the stability of FMDV vaccine antigens, including targeted capsid mutations and formulation-based stabilizers [28,29,30,31,32,33,34,35,36]. Mutation-based approaches can enhance capsid thermostability; however, amino acid substitutions near surface-exposed epitopes may alter antigenicity and affect vaccine matching, particularly in serotype O viruses that exhibit substantial antigenic diversity [33]. Formulation stabilizers can reduce antigen degradation during storage; however, they require optimization of formulation conditions and cannot fully overcome the inherent instability of the viral capsid [37,38].

The VP1 substitution strategy used in this study provides a genetic approach that preserves antigenicity, while improving stability. By introducing the immunodominant VP1 region of the O JC strain into the structurally stable capsid backbone, we improved the stability of 146S particles without compromising immunogenicity. This design maintained the antigenic properties of the O JC strain while enhancing the physical stability of the vaccine antigen.

This strategy is also advantageous for vaccine manufacturing. The use of a well-characterized antigen backbone, which has already been widely adopted in FMD vaccine production, facilitates integration into established vaccine manufacturing processes. Improved capsid stability may enhance antigen recovery during purification, reduce degradation during storage and transport, and improve batch-to-batch consistency during large-scale vaccine production. Additionally, this approach offers advantages for vaccine development against newly emerging FMDV strains. When a new vaccine strain is required but the candidate virus is physically unstable, the VP1 sequence alone may be sufficient to generate a stable recombinant vaccine antigen. This has enabled the rapid development of vaccine strains based primarily on VP1 sequence information, particularly for viruses with a high risk of introduction.

Several studies have reported that chimeric FMDV with altered immunogenic properties can be generated by substituting the VP1 region, particularly the GH loop [39,40,41,42,43]. However, these approaches have mainly focused on antigenic matching or enhancing cross-protective immune responses, without considering the physical stability of the vaccine strains. Here, we extended this strategy by applying a VP1-based chimeric design to enhance the physical stability of FMDV vaccine antigens while preserving their antigenicity. This approach provides a practical strategy for expanding the use of highly antigenic but physically labile viruses in vaccine development and may facilitate the rapid generation of stable vaccine candidates against newly emerging FMDV strains. These findings highlight the potential of VP1-based design to simultaneously optimize antigenicity and stability, offering advantages across vaccine manufacturing, storage, and distribution, and ultimately supporting the development of more stable and effective FMD vaccines.

## 5. Conclusions

In this study, we demonstrated that substitution of the VP1 structural protein from the antigenically relevant O JC strain with an O1 M backbone significantly improved capsid stability while preserving immunogenicity. The resulting chimeric virus exhibited enhanced resistance to thermal stress, chloroform exposure, and prolonged storage while maintaining strong antibody responses in vaccinated pigs. These findings indicate that VP1-directed chimeric engineering is a practical strategy for improving the stability of inactivated FMDV vaccine antigens. This approach may facilitate the development of stable vaccine strains against emerging FMDV variants.

## Data Availability

All data generated or analyzed during the study are included in this published article. The dataset used during the current study is available from the corresponding author on reasonable request.

## Supplementary Materials

The following supporting information can be downloaded at: https://www.mdpi.com/article/10.3390/vaccines14050371/s1, Figure S1: Comparative analysis of the thermal stability of chimeric FMDV strains.
