# Supplementary material for: Enhancing Capsid Stability of a Foot-and-Mouth Disease Virus Vaccine Strain Through VP1-Directed Chimeric Design While Preserving Antigenicity
Source: Vaccines (Basel). 2026 Apr 22;14(5):371. doi: 10.3390/vaccines14050371 (PMC13211603; doi:10.3390/vaccines14050371)
Supplement: Supplementary file 1 [file vaccines-14-00371-s001.zip › vaccines-4258582-supplementary.pdf]

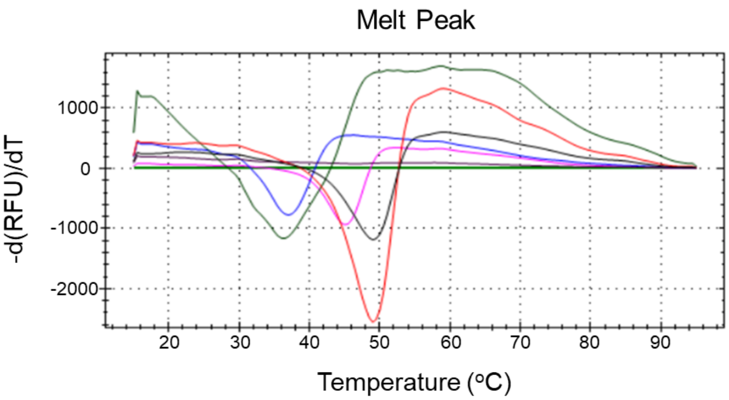

Melt curve summary

Sample	Melt Temperature (T_m, °C)
O JC wild	36.5
O PA2 wild	49.0
(R) O PA2–O JC_VP1	49.0
(R) O PA2–O JC_VP2	37.0
(R) O PA2–O JC_VP3	45.0
(R) O PA2–O JC_P1	None

Supplementary Figure S1. Comparative analysis of the thermal stability of chimeric FMDV strains. Each of the three capsid proteins (VP1, VP2, and VP3) from the unstable O JC strain was individually substituted into the O PanAsia2 (PA2) backbone to assess their effect on virus particle stability. Thermal stability of the resulting chimeric viruses was evaluated using a particle stability thermal release assay (PaSTRy) to determine the melting temperature (T_m, °C) of purified 146S antigens.
